# Supplementary material for: Multifaceted Antibiotic Resistance in Diabetic Foot Infections: A Systematic Review
Source: Microorganisms. 2025 Oct 6;13(10):2311. doi: 10.3390/microorganisms13102311 (PMC12566198; doi:10.3390/microorganisms13102311)
Supplement: Supplementary file 1 [file microorganisms-13-02311-s001.zip › microorganisms-3659207-supplementary.pdf]

Supplementary: PRISMA Checklist

Section and Topic	Item #	Checklist item	Reported (Yes/No)
**TITLE**			
Title	1	Identify the report as a systematic review.	Yes
**BACKGROUND**			
Objectives	2	Provide an explicit statement of the main objective(s) or question(s) the review addresses.	Yes
**METHODS**			
Eligibility criteria	3	Specify the inclusion and exclusion criteria for the review.	Yes
Information sources	4	Specify the information sources (e.g. databases, registers) used to identify studies and the date when each was last searched.	Yes
Risk of bias	5	Specify the methods used to assess risk of bias in the included studies.	Yes
Synthesis of results	6	Specify the methods used to present and synthesise results.	Yes
**RESULTS**			
Included studies	7	Give the total number of included studies and participants and summarise relevant characteristics of studies.	Yes
Synthesis of results	8	Present results for main outcomes, preferably indicating the number of included studies and participants for each. If meta-analysis was done, report the summary estimate and confidence/credible interval. If comparing groups, indicate the direction of the effect (i.e. which group is favoured).	Yes
**DISCUSSION**			
Limitations of evidence	9	Provide a brief summary of the limitations of the evidence included in the review (e.g. study risk of bias, inconsistency and imprecision).	Yes
Interpretation	10	Provide a general interpretation of the results and important implications.	Yes
**OTHER**			
Funding	11	Specify the primary source of funding for the review.	NA
Registration	12	Provide the register name and registration number.	NA

From: Page MJ, McKenzie JE, Bossuyt PM, Boutron I, Hoffmann TC, Mulrow CD, et al. The PRISMA 2020 statement: an updated guideline for reporting systematic reviews. BMJ 2021;372:n71. doi: 10.1136/bmj.n71. This work is licensed under CC BY 4.0. To view a copy of this license, visit https://creativecommons.org/licenses/by/4.0/

Supplementary Table S1: Quality Assessment of the included studies (Risk of Bias Analysis using modified Cochrane ROBINS E tool)

Quality Category	Questions	Perim, Michele Cezimbra, et al. [29]	Boschetti, Giovanni, et al. [25]	Bouharkat,B.,et al. [21]	Ismail,A. A., et al. [20]	Sannathimmappa,M.B. ,et al. [19]	Demetriou M, et al. [22]	Caruso, P.,et al. [1]	Goh,T. C.,et al. [28]	Le, Tan To Anh,et al. [5]	Alvaro-Afonso,Francisco Javier, et al. [27]	Sanchez, C.A., et al.	Najari, H.R.,et al. [7]	Khan, M.S.,et al. [2]	Moya-Salazar, J.,et al. [18]	Jouhar L, et al. [9]	Yan X, et al. [26]	Hatipoglu, M.,et al. [10]	Sekhar, S., et al. [3]	Xiaoying Xie, et al. [23]	Pontes DÊnisson Guedes, et al. [12]	Sanchez-Sanchez M, et al.	Ji X, et al. [15]	Man Wu 1, et al. [16]F	Asegdew Atlaw, 1,2, et al. [17]	Saseedharan,S., et al. [4]	Pessoae Costa T, et al. [24]	Kow, R Y et al. [14]	Li, Xiangyan et al. [11]
**Quality Assessment Tool Developed for Observational Cohort Studies by the authors**																													
Research question	Did the study report clear aims (including all of the following: population, exposure, and outcome of interest)?	Y	Y	Y	Y	Y	Y	Y	Y	Y	Y	Y	Y	Y	Y	Y	Y	Y	Y	Y	Y	Y	Y	Y	Y	Y	Y	Y	Y
	Was the study setting defined (eg, hospital based; single centre or multicentre)?	Y	Y	Y	Y	Y	Y	Y	Y	N	Y	Y	Y	Y	Y	Y	Y	Y	Y	Y	Y	Y	Y	Y	Y	Y	Y	Y	Y
	Was the study reported as a prospective study (eg, hypothesis/research question defined before recruitment of patients)	Y	N	N	N	N	N	N	Y	N	N	N	N	Y	N	N	N	Y	N	Y	Y	N	N	N	N	Y	N	N	N
Selection criteria	Were the methods for recruitment /sampling detailed in the study?	Y	Y	Y	Y	Y	N	Y	N	Y	N	Y	Y	Y	Y	N	Y	Y	Y	Y	Y	Y	Y	Y	Y	Y	Y	Y	Y
	Was the diagnosis of DFI and DFU adequately defined (ie, positive diagnosis of DFI by one or several of the following: presence of DM type 1 or 2 with presence of ulcer; discontinuation of the epithelium, infection: local signs of infection or systemic features of infection or gangrene; presence of black necrotic tissue with clinical examination or presence of OM;	N	Y	Y	Y	N	Y	Y	Y	Y	Y	N	Y	Y	Y	Y	Y	Y	Y	Y	Y	N	Y	Y	Y	N	Y	N	Y

	probing to bone or medical imaging with or without biochemical confirmation of infection)																												
	Confirmation of an index admission following a DFI	N	N	N	N	N	N	Y	N	N	N	N	Y	N	N	N	N	Y	N	N	N	Y	Y	Y	N	N	Y	Y	Y
	Were inclusion and exclusion criteria detailed?	N	Y	Y	Y	N	Y	Y	Y	Y	Y	Y	N	Y	Y	N	Y	Y	N	N	N	N	Y	N	Y	N	Y	Y	Y
Sub total		4/7	5/7	5/7	5/7	3/7/	4/7	6/7	5/7	4/7	4/7	4/7	5/7	6/7	5/7	3/7	5/7	7/7	4/7	5/7	5/7	4/7	6/7	5/7	5/7	4/7	6/7	5/7	6/7
Participa nt character istics	Was DFI severity defined in the cases? (ie, based on Wagner, SINBAD, Texas or any similar DFI classifications)	N	Y	N	Y	N	Y	Y	Y	Y	Y	Y	Y	Y	N	Y	Y	Y	Y	Y	Y	Y	Y	Y	Y	Y	Y	N	Y
	Sufficient relevant baseline medical and demographic information characterizing participants was provided (or reference to previously published baseline data provided). Defined as including ≥5 of the following: age, gender, ethnicity, smoking, PN, HTN, ABI, DM, previous AMI/CAD, PN, renal impairment.	N	N	N	Y	Y	Y	Y	Y	Y	Y	N	Y	Y	N	Y	Y	Y	Y	Y	Y	Y	Y	Y	Y	N	Y	N	Y
Sample size	Was the sample size >100 participants?	N	Y	Y	Y	Y	Y	Y	Y	Y	Y	Y	Y	Y	Y	Y	Y	Y	Y	Y	Y	Y	Y	N	Y	Y	N	Y	Y
	Was the basis of sample size/calculation reported in methodology?	N	N	N	N	N	N	N	N	N	N	N	N	Y	N	N	N	N	Y	N	N	N	N	N	N	N	N	N	N
Sub total		0/4	2/4	1/4	3/4	2/4	3/4	3/4	3/4	3/4	3/4	2/4	3/4	4/4	1/4	3/4	3/4	3/4	4/4	3/4	3/4	3/4	3/4	2/4	3/4	2/4	2/4	1/4	3/4
Outcome	Was the primary outcome defined as readmission to a hospital as an inpatient ≤30 days after the index DFI related admission?	N	N	N	N	N	N	N	N	N	N	N	N	N	N	N	N	N	N	N	N	N	N	N	N	N	N	N	N
	Was the indication for readmission noted	N	N	N	N	N	N	N	N	N	N	N	N	N	N	N	N	N	N	N	N	N	N	N	N	N	N	N	N
	Was it reported whether readmission was	N	N	N	N	N	N	N	N	N	N	N	N	N	N	N	N	N	N	N	N	N	N	N	N	N	N	N	N

	planned or unplanned																												
Methods	Process by which follow- up was performed to identify outcomes described (eg, ICD 9/10 codes, dataset, hospital records, prospective phone calls, patient reports etc)	N	N	N	N	N	N	Y	Y	N	Y	Y	Y	N	N	N	Y	Y	Y	N	Y	Y	Y	N	Y	Y	Y	Y	N
	Ethical issues (eg, consent, patient confidentiality, ethics approval) addressed	Y	N	Y	Y	Y	Y	Y	Y	Y	Y	Y	Y	Y	Y	Y	Y	Y	Y	Y	Y	N	Y	N	Y	Y	Y	N	Y
Analysis	Did the study exclude or adjust for ≥2 confounders using one or several statistical methods (eg, logistic regression): age, gender, HTN, IHD, ABI, BMI, DM, smoking, dyslipidaemia impaired renal function, previous stroke, preoperative wound, ASA classification vs readmissions	N	N	N	N	N	N	N	N	N	N	N	N	N	N	N	Y	N	N	N	N	N	Y	N	N	N	Y	N	N
	Measure of association (eg, odds ratio) included for each risk factor	N	N	N	N	Y	N	Y	N	Y	N	N	N	Y	N	N	Y	N	N	N	N	N	Y	N	N	N	N	N	N
	Whether the association was statistically significant (eg, *P* value) included	N	N	Y	N	N	N	Y	N	Y	N	N	Y	Y	N	Y	Y	Y	N	Y	N	N	Y	Y	N	N	Y	N	Y
	Did the study report findings in the context of the existing literature?	Y	Y	Y	Y	Y	Y	Y	Y	N	Y	Y	Y	Y	Y	Y	Y	Y	Y	Y	Y	Y	Y	Y	Y	Y	Y	Y	Y
Sub total		2/9	1/9	3/9	2/9	3/9	2/9	5/9	3/9	3/9	3/9	3/9	4/9	4/9	2/9	3/9	6/9	4/9	3/9	3/9	3/9	2/9	6/9	2/9	3/9	3/9	5/9	2/9	3/9
Individual score (Y)		6/20	8/20	9/20	10/20	8/20	9/20	14/20	11/20	10/20	10/20	9/20	12/20	14/20	8/20	9/20	14/20	14/20	11/20	11/20	11/20	9/20	15/20	9/20	11/20	9/20	13/20	8/20	12/20
Averaged Score (%)		30%	40%	45%	50%	40%	45%	70%	55%	50%	50%	45%	60%	70%	40%	45%	70%	70%	55%	55%	55%	45%	75%	45%	55%	45%	65%	40%	60%

Supplementary tables

Supplementary Table S2: Summary of the strongest and significant correlations (|r| > 0.4, *p* < 0.05) between comorbidities and resistance variables in *Staphylococcus aureus*:

	Comorbidity	Resistance	Correlation	P-value
4	Dyslipidemia	SA_vs_clindamycin	0.9965143429	0.0002469071
5	Dyslipidemia	SA_vs_gentamicin	0.9863509315	0.0019102809
3	Dyslipidemia	SA_vs_vancomycin	0.9784060921	0.0037968111
2	Dyslipidemia	SA_vs_erythromycin	0.9734224231	0.0051804555
1	Dyslipidemia	SA_vs_penicillin	0.9492082327	0.0136360039
0	HTN	SA_vs_erythromycin	0.8825777721	0.00474414797

Supplementary Table S3: Summary of the strongest and significant correlations (|r| > 0.4, *p* < 0.05) between comorbidities and resistance variables in *Enterococcus spp*:

	Comorbidity	Resistance	Correlation	P-value
25	Current_Smoker	E_vs_vancomycin	0.9965232937	0.0034767063
5	HTN	E_vs_tetracycline	0.9930374976	0.0069625024
3	HTN	E_vs_vancomycin	0.9923272879	0.0076727121
6	HTN	E_vs_gentamicin	0.9901215431	0.0098784569
1	HTN	E_vs_ampicillin	0.989121689	0.010878311
27	Current_Smoker	E_vs_tetracycline	0.9883702606	0.0116297394
0	HTN	E_vs_penicillin	0.9781016416	0.0218983584
28	Current_Smoker	E_vs_gentamicin	0.9748794339	0.0251205661
23	Current_Smoker	E_vs_ampicillin	0.9635664884	0.0364335116
46	Nephropathy	E_vs_erythromycin	0.9558444286	0.0441555714
4	HTN	E_vs_quinolones	0.94836289	0.05163711
22	Current_Smoker	E_vs_penicillin	0.9448455149	0.0551544851
2	HTN	E_vs_erythromycin	0.9369581818	0.0630418182
26	Current_Smoker	E_vs_quinolones	0.9164235926	0.0835764074
18	PAD	E_vs_quinolones	0.9052031887	0.0947968113
44	Nephropathy	E_vs_penicillin	0.9018310356	0.0981689644
24	Current_Smoker	E_vs_erythromycin	0.8868558897	0.1131441103
45	Nephropathy	E_vs_ampicillin	0.8712935793	0.1287064207
20	PAD	E_vs_gentamicin	0.8564148033	0.1435851967
10	Cardiovascular_Disease	E_vs_erythromycin	0.8551861105	0.1448138895
19	PAD	E_vs_tetracycline	0.8412916229	0.1587083771
21	PAD	E_vs_aminoglycosides	0.8290721288	0.1709278712
17	PAD	E_vs_vancomycin	0.8051569477	0.1948430523
8	Cardiovascular_Disease	E_vs_penicillin	0.7668428809	0.2331571191
47	Nephropathy	E_vs_vancomycin	0.7536756621	0.2463243379
36	Previous_Amputation	E_vs_vancomycin	0.7444116816	0.2555883184
9	Cardiovascular_Disease	E_vs_ampicillin	0.7221027954	0.2778972046
49	Nephropathy	E_vs_tetracycline	0.7202422406	0.2797577594
32	Previous_Ulceration	E_vs_erythromycin	0.7169166266	0.2830833734

	Comorbidity	Resistance	Correlation	P-value
38	Previous_Amputation	E_vs_tetracycline	0.7082828752	0.2917171248
50	Nephropathy	E_vs_gentamicin	0.7015304522	0.2984695478
15	PAD	E_vs_ampicillin	0.6790675591	0.3209324409
39	Previous_Amputation	E_vs_gentamicin	0.6646946749	0.3353053251
30	Previous_Ulceration	E_vs_penicillin	0.6428570396	0.3571429604
34	Previous_Amputation	E_vs_ampicillin	0.6412463278	0.3587536722
14	PAD	E_vs_penicillin	0.6300349828	0.3699650172
33	Previous_Amputation	E_vs_penicillin	0.6078038384	0.3921961616
31	Previous_Ulceration	E_vs_ampicillin	0.6053506878	0.3946493122
7	HTN	E_vs_aminoglycosides	0.5978322133	0.4021677867
48	Nephropathy	E_vs_quinolones	0.5906903795	0.4093096205
37	Previous_Amputation	E_vs_quinolones	0.5635552769	0.4364447231
11	Cardiovascular_Disease	E_vs_vancomycin	0.5625650191	0.4374349809
12	Cardiovascular_Disease	E_vs_tetracycline	0.5286805326	0.4713194674
29	Current_Smoker	E_vs_aminoglycosides	0.5249177441	0.4750822559
35	Previous_Amputation	E_vs_erythromycin	0.5221523981	0.4778476019
13	Cardiovascular_Disease	E_vs_gentamicin	0.5144957554	0.4855042446
16	PAD	E_vs_erythromycin	0.5087919247	0.4912080753
43	Dyslipidemia	E_vs_vancomycin	-0.5496565719	0.4503434281
41	Dyslipidemia	E_vs_ampicillin	-0.6948437388	0.3051562612
40	Dyslipidemia	E_vs_penicillin	-0.7378949061	0.2621050939

Supplementary Table S4: Summary of the strongest and significant correlations (|r| > 0.4, $p < 0.05$) between comorbidities and resistance variables in *Pseudomonas aeruginosa*:

	Comorbidity	Resistance	Correlation	P-value
14	Neuropathy	P_vs_cephalosporins	1	0
18	Neuropathy	P_vs_carbapenems	1	0
19	Neuropathy	P_vs_aminoglycosides	1	0
16	Neuropathy	P_vs_tetracycline	0.9994597806	0.0000150714

	Comorbidity	Resistance	Correlation	P-value
17	Neuropathy	P_vs_gentamicin	0.9522153969	0.0124488212
25	Current_Smoker	P_vs_piperacillin_tazo	0.9454503978	0.015168247
26	Current_Smoker	P_vs_imipenem	0.9178690251	0.0279042507
38	Nephropathy	P_vs_piperacillin_tazo	0.8714183826	0.0542679487
20	PAD	P_vs_erythromycin	0.8609800513	0.0609087104
15	Neuropathy	P_vs_quinolones	0.8520240708	0.0667940747
2	HTN	P_vs_piperacillin_tazo	0.8516487707	0.0670443746
21	PAD	P_vs_amox_clav	0.8321073786	0.0804692597
22	PAD	P_vs_clindamycin	0.8321073786	0.0804692597
7	HTN	P_vs_tetracycline	0.8223806538	0.0874270989
9	HTN	P_vs_carbapenems	0.8081859089	0.0978918904
10	HTN	P_vs_aminoglycosides	0.8081859089	0.0978918904
5	HTN	P_vs_cephalosporins	0.8081859089	0.0978918904
39	Nephropathy	P_vs_imipenem	0.7989720905	0.1048741995
23	Current_Smoker	P_vs_ampicillin	0.747718811	0.1462185067
4	HTN	P_vs_imipenem	0.7249141018	0.1658632204
12	Neuropathy	P_vs_piperacillin_tazo	0.7174657014	0.1724327975
8	HTN	P_vs_gentamicin	0.7169146772	0.1729217168
11	Neuropathy	P_vs_ampicillin	0.6711225198	0.2148847092
13	Neuropathy	P_vs_imipenem	0.6527552448	0.2324130433
28	Current_Smoker	P_vs_quinolones	0.6498192486	0.2352497234
30	Current_Smoker	P_vs_gentamicin	0.6323861906	0.2522838866
0	HTN	P_vs_ampicillin	0.6047630884	0.2799162151
6	HTN	P_vs_quinolones	0.6037774868	0.280916099
37	Nephropathy	P_vs_ampicillin	0.5947675382	0.2900997349
29	Current_Smoker	P_vs_tetracycline	0.5796175593	0.3057132628
27	Current_Smoker	P_vs_cephalosporins	0.5761989904	0.3092654698
24	Current_Smoker	P_vs_erythromycin	0.5761989904	0.3092654698
31	Current_Smoker	P_vs_carbapenems	0.5761989904	0.3092654698
32	Current_Smoker	P_vs_aminoglycosides	0.5761989904	0.3092654698

	Comorbidity	Resistance	Correlation	P-value
33	Previous_Amputation	P_vs_piperacillin_tazo	0.5746446601	0.3108840365
34	Dyslipidemia	P_vs_ampicillin	-0.5161266033	0.3733170641
1	HTN	P_vs_amox_clav	-0.5331397894	0.3548761302
3	HTN	P_vs_clindamycin	-0.5331397894	0.3548761302
36	Dyslipidemia	P_vs_quinolones	-0.5618911367	0.3242454123
35	Dyslipidemia	P_vs_erythromycin	-0.6123724357	0.2722284012

Supplementary Table S5: Summary of the strongest and significant correlations (|r| > 0.4, *p* < 0.05) between comorbidities and resistance variables in *Escherichia coli*:

	Comorbidity	Resistance	Correlation	P-value
47	Current_Smoker	E_vs_piperacillin_tazo	0.9927198688	0.0007448483
48	Current_Smoker	E_vs_imipenem	0.9927198688	0.0007448483
49	Current_Smoker	E_vs_cephalosporins	0.9927198688	0.0007448483
50	Current_Smoker	E_vs_quinolones	0.9927198688	0.0007448483
44	Current_Smoker	E_vs_penicillin	0.9921125392	0.0008398954
52	Current_Smoker	E_vs_gentamicin	0.9905275927	0.0011051122
46	Current_Smoker	E_vs_amox_clav	0.9851676048	0.002163626
51	Current_Smoker	E_vs_tetracycline	0.9798208441	0.0034305947
83	Nephropathy	E_vs_aminoglycosides	0.9682458366	0.0067601355
45	Current_Smoker	E_vs_erythromycin	0.9599881986	0.009549754
8	HTN	E_vs_tetracycline	0.9269899515	0.0234203616
71	Previous_Amputation	E_vs_tetracycline	0.9261048864	0.0238442758
82	Nephropathy	E_vs_carbapenems	0.9185586535	0.0275564637
9	HTN	E_vs_gentamicin	0.8959361412	0.0396630222
72	Previous_Amputation	E_vs_gentamicin	0.886243107	0.0452634404
0	HTN	E_vs_penicillin	0.8839002794	0.0466518761

	Comorbidity	Resistance	Correlation	P-value
62	Previous_Ulceration	E_vs_tetracycline	0.8777288587	0.0503720747
42	PAD	E_vs_tetracycline	0.875526315	0.0517214449
64	Previous_Amputation	E_vs_penicillin	0.8713141524	0.0543330606
6	HTN	E_vs_cephalosporins	0.8669353321	0.0570905215
7	HTN	E_vs_quinolones	0.8669353321	0.0570905215
4	HTN	E_vs_piperacillin_tazo	0.8669353321	0.0570905215
5	HTN	E_vs_imipenem	0.8669353321	0.0570905215
70	Previous_Amputation	E_vs_quinolones	0.8506015515	0.0677443391
69	Previous_Amputation	E_vs_cephalosporins	0.8506015515	0.0677443391
68	Previous_Amputation	E_vs_imipenem	0.8506015515	0.0677443391
67	Previous_Amputation	E_vs_piperacillin_tazo	0.8506015515	0.0677443391
3	HTN	E_vs_amox_clav	0.835307719	0.0782191401
66	Previous_Amputation	E_vs_amox_clav	0.8344009875	0.078854651
63	Previous_Ulceration	E_vs_gentamicin	0.8284410852	0.0830710171
43	PAD	E_vs_gentamicin	0.8258686525	0.0849116585
65	Previous_Amputation	E_vs_erythromycin	0.8236594683	0.0865022408
55	Previous_Ulceration	E_vs_penicillin	0.8105096535	0.096154171
35	PAD	E_vs_penicillin	0.8078199998	0.0981663827
2	HTN	E_vs_erythromycin	0.7924914303	0.1098716082
61	Previous_Ulceration	E_vs_quinolones	0.7859777654	0.1149644882
60	Previous_Ulceration	E_vs_cephalosporins	0.7859777654	0.1149644882
59	Previous_Ulceration	E_vs_imipenem	0.7859777654	0.1149644882
58	Previous_Ulceration	E_vs_piperacillin_tazo	0.7859777654	0.1149644882
20	Retinopathy	E_vs_carbapenems	0.7843821125	0.1162226214
38	PAD	E_vs_piperacillin_tazo	0.7831394648	0.1172052592
39	PAD	E_vs_imipenem	0.7831394648	0.1172052592
40	PAD	E_vs_cephalosporins	0.7831394648	0.1172052592
41	PAD	E_vs_quinolones	0.7831394648	0.1172052592
57	Previous_Ulceration	E_vs_amox_clav	0.7675771527	0.1297177817
37	PAD	E_vs_amox_clav	0.7646543747	0.1321095279

	Comorbidity	Resistance	Correlation	P-value
56	Previous_Ulceration	E_vs_erythromycin	0.7587250488	0.1370010941
36	PAD	E_vs_erythromycin	0.7558814537	0.1393655565
21	Retinopathy	E_vs_aminoglycosides	0.7553201374	0.1398337017
17	Hypercholesterolemia	E_vs_tetracycline	0.7351470441	0.1569592001

Supplementary Table S6: Summary of the strongest and significant correlations (|r| > 0.4, $p < 0.05$) between comorbidities and resistance variables in *Proteus spp*:

	Comorbidity	Resistance	Correlation	P-value
42	Current_Smoker	P_vs_erythromycin	0.9992788935	0.0000232427
43	Current_Smoker	P_vs_amox_clav	0.9989130825	0.0000430089
44	Current_Smoker	P_vs_piperacillin_tazo	0.9989130825	0.0000430089
45	Current_Smoker	P_vs_imipenem	0.9989130825	0.0000430089
46	Current_Smoker	P_vs_cephalosporins	0.9989130825	0.0000430089
47	Current_Smoker	P_vs_quinolones	0.9989130825	0.0000430089
41	Current_Smoker	P_vs_penicillin	0.9983377316	0.000081335
50	Former_Smoker	P_vs_tetracycline	0.9982652373	0.0000867123
69	Previous_Amputation	P_vs_gentamicin	0.9975856684	0.0001423547
48	Current_Smoker	P_vs_gentamicin	0.9903258021	0.0011405783
62	Previous_Amputation	P_vs_penicillin	0.9886141226	0.00145593
63	Previous_Amputation	P_vs_erythromycin	0.9854602021	0.0021000129
72	Nephropathy	P_vs_aminoglycosides	0.9798501839	0.0034231307
64	Previous_Amputation	P_vs_amox_clav	0.9675769477	0.0069741486
65	Previous_Amputation	P_vs_piperacillin_tazo	0.9675769477	0.0069741486
66	Previous_Amputation	P_vs_imipenem	0.9675769477	0.0069741486
67	Previous_Amputation	P_vs_cephalosporins	0.9675769477	0.0069741486
68	Previous_Amputation	P_vs_quinolones	0.9675769477	0.0069741486
49	Former_Smoker	P_vs_ampicillin	0.9092074297	0.0323895253

	Comorbidity	Resistance	Correlation	P-value
7	HTN	P_vs_gentamicin	0.8508153944	0.067601222
39	PAD	P_vs_tetracycline	0.8297886954	0.0821117342
0	HTN	P_vs_penicillin	0.8105972874	0.0960888232
60	Previous_Ulceration	P_vs_gentamicin	0.8027950985	0.1019593938
1	HTN	P_vs_erythromycin	0.8000687802	0.1040354985
70	Dyslipidemia	P_vs_ampicillin	0.7788910196	0.1205834079
52	Previous_Ulceration	P_vs_penicillin	0.7515602913	0.1429813443
2	HTN	P_vs_amox_clav	0.7513470877	0.1431604493
3	HTN	P_vs_piperacillin_tazo	0.7513470877	0.1431604493
4	HTN	P_vs_imipenem	0.7513470877	0.1431604493
5	HTN	P_vs_cephalosporins	0.7513470877	0.1431604493
6	HTN	P_vs_quinolones	0.7513470877	0.1431604493
17	Hypercholesterolemia	P_vs_gentamicin	0.7479031139	0.1460627121
27	Retinopathy	P_vs_gentamicin	0.7479031139	0.1460627121
53	Previous_Ulceration	P_vs_erythromycin	0.738427987	0.1541347465
37	Cardiovascular_History	P_vs_gentamicin	0.7053798788	0.1832467676
9	Hypercholesterolemia	P_vs_penicillin	0.6913482681	0.196033542
19	Retinopathy	P_vs_penicillin	0.6913482681	0.196033542
38	PAD	P_vs_ampicillin	0.6886763931	0.198495853
58	Previous_Ulceration	P_vs_quinolones	0.678801123	0.207671004
57	Previous_Ulceration	P_vs_cephalosporins	0.678801123	0.207671004
56	Previous_Ulceration	P_vs_imipenem	0.678801123	0.207671004
55	Previous_Ulceration	P_vs_piperacillin_tazo	0.678801123	0.207671004
54	Previous_Ulceration	P_vs_amox_clav	0.678801123	0.207671004
20	Retinopathy	P_vs_erythromycin	0.6769901775	0.209366107
10	Hypercholesterolemia	P_vs_erythromycin	0.6769901775	0.209366107
29	Cardiovascular_History	P_vs_penicillin	0.6520405498	0.2331026943
30	Cardiovascular_History	P_vs_erythromycin	0.6384988115	0.2462744143
11	Hypercholesterolemia	P_vs_amox_clav	0.6123724357	0.2722284012
71	Nephropathy	P_vs_carbapenems	0.6123724357	0.2722284012

	Comorbidity	Resistance	Correlation	P-value
22	Retinopathy	P_vs_piperacillin_tazo	0.6123724357	0.2722284012

Supplementary Figure

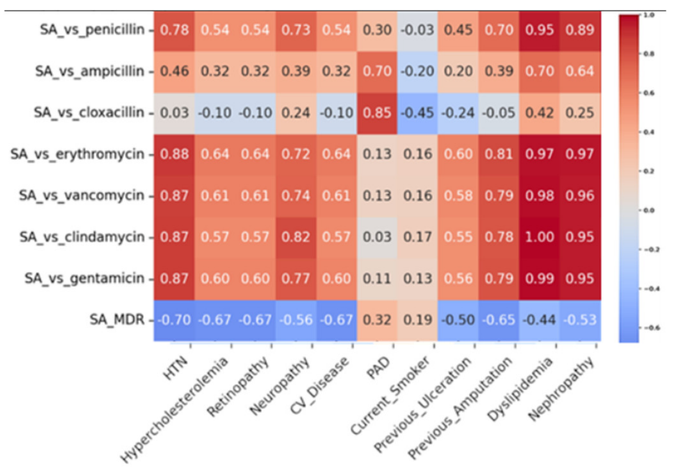

(a)

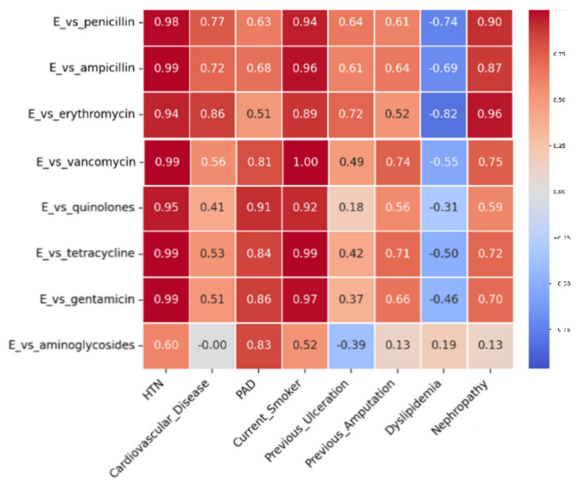

(b)

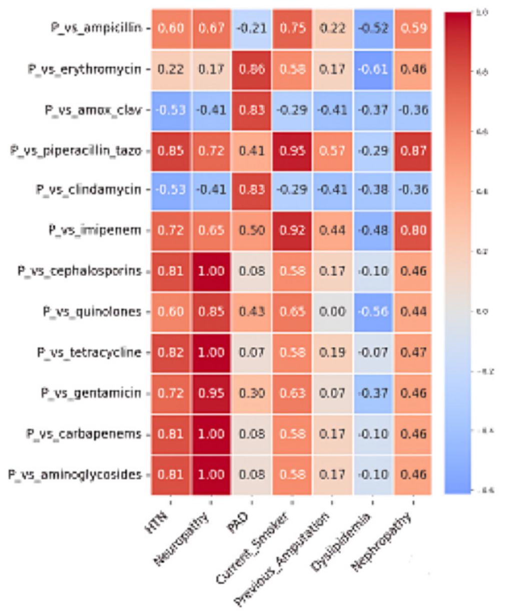

(c)

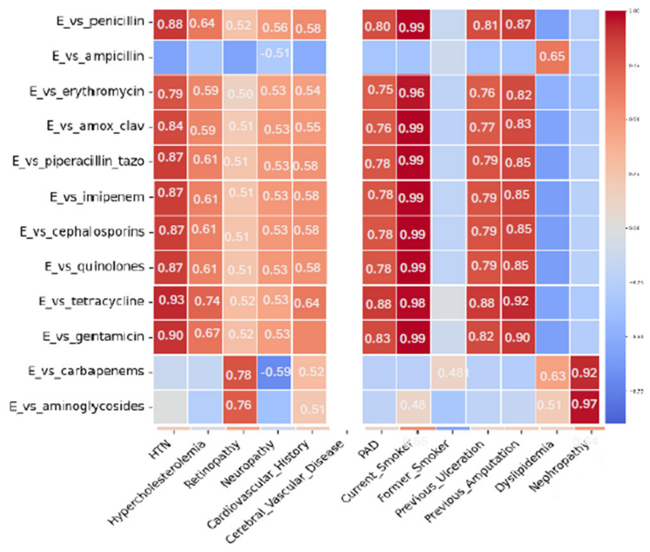

(d)

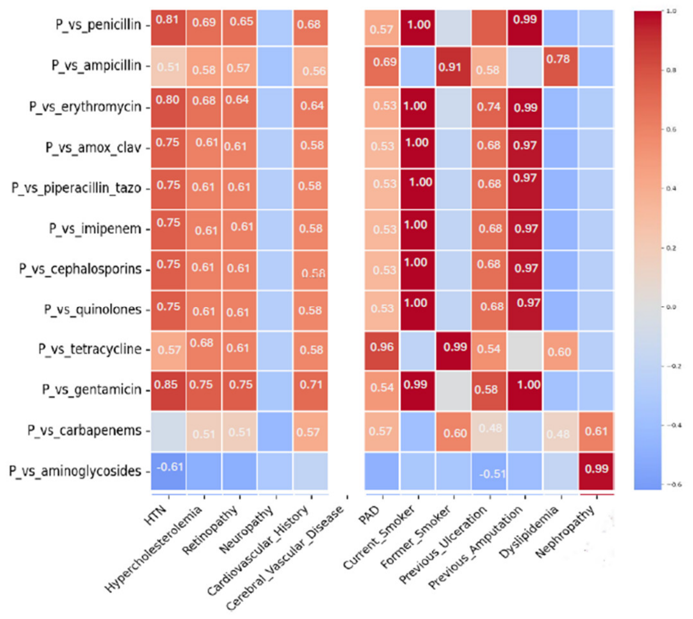

(**e**)

Figure S1. Correlation Heatmap between Comorbidities and Antibiotic Resistance patterns in (A) *S. aureus*, (B) Enterococcus spp, (C) *P. aeruginosa*, (D) *E. coli* and (E) Proteus spp.
